# The Induction with Foley OR Misoprostol (INFORM) Study dataset. A dataset of 602 women with hypertensive disease in pregnancy, in India, randomised to either Foley catheter or oral misoprostol for induction of labour

**DOI:** 10.1186/s13104-021-05772-9

**Published:** 2021-09-10

**Authors:** Shuchita Mundle, Hillary Bracken, Vaishali Khedikar, Jayashree Mulik, Brian Faragher, Thomas Easterling, Simon Leigh, Paul Granby, Alan Haycox, Mark A. Turner, Kate Lightly, Miroslava Ebringer, Zarko Alfirevic, Beverly Winikoff, Andrew D. Weeks

**Affiliations:** 1 Department of Obstetrics and Gynecology, All India Institute of Medical Sciences, Nagpur, India; 2 Gynuity Health Projects (GHP), 220 East 42nd Street, New York, NY 10017 USA; 3 Daga Memorial Women’s Government Hospital, Nagpur, India 440018; 4 Government Medical College, Nagpur, 440003 India; 5 Liverpool School of Tropical Medicine, Pembroke Place, Liverpool, L3 5QA UK; 6 University of Washington, Seattle, Washington 98195 USA; 7 Nexus Clinical Analytics, Euxton, PR7 6BX Lancashire UK; 8 Certus Analytics, Formby, Liverpool, L37 2LR UK; 9 University of Liverpool Management School, Chatham Street, Liverpool, L69 7ZH UK; 10 University of Liverpool and Liverpool Women’s Hospital for Liverpool Health Partners, Crown Street, Liverpool, L8 7SS UK; 11 Gynuity Health Projects (GHP), 220 East 42nd Street, New York, NY 10017 USA

**Keywords:** Induction, Labour, Hypertension, Pre-eclampsia, Misoprostol, Foley catheter, Dataset

## Abstract

**Objectives:**

Induction of labour (IOL), or starting labour artificially, can be a lifesaving intervention for pregnant women and their babies, and rates are rising significantly globally. As rates increase, it becomes increasingly important to fully evaluate all available data, especially that from low income settings where the potential benefits and harms are greater. The goal of this paper is to describe the datasets collected as part of the Induction with Foley OR Misoprostol (INFORM) Study, a randomised trial comparing two of the recommended methods of cervical ripening for labour induction, oral misoprostol and Foley catheter, in women being induced for hypertension in pregnancy, at two sites in India during 2013–15.

**Data description:**

This dataset includes comprehensive data on 602 women who underwent IOL for hypertensive disorders in pregnancy. Women were randomly assigned to cervical ripening with oral misoprostol or a transcervical Foley catheter in two government hospitals in India. The main dataset has 367 variables including monitoring during the induction of labour, medications administered, timing and mode of delivery, measures of neonatal morbidity and mortality, maternal mortality and morbidity, maternal satisfaction and health economic data. The dataset is anonymised and available on ReShare.

## Objectives

IOL rates are rising rapidly around the world, both in high and low income settings. High quality IOL studies from low income settings are rare, and most are undertaken in high income settings. However, the findings of high income setting studies may not be applicable to resource constrained settings where the risks and benefits of induction are far higher for patients, and adequate resources are often not available. Pre-eclampsia is one of the most prevalent causes of morbidity and mortality around the world. This dataset, which also includes health economic data, is important for researchers, clinicians and policy makers investigating IOL, maternity care in high and low income settings and pre-eclampsia.

Low dose oral misoprostol and the Foley catheter are low cost cervical ripening methods recommended for use around the world including low income settings, where pre-eclampsia causes the most significant burden [1]. However few studies have directly compared them [2, 3]. Few similar datasets of pre-eclamptic patients and IOL patients are currently available. The INFORM study was undertaken at two public hospitals in Nagpur, India, between December 2013 and June 2015. The aims of this randomised controlled trial were “to directly compare the efficacy, safety, acceptability and cost effectiveness of misoprostol and the Foley catheter in the induction of labour among women with gestational hypertension, in a low resource setting” [4, 5].

The study protocol [4], clinical results [5] and health economic analysis [6] have been published previously, but the dataset was not published at that time due to concerns that some variables could potentially compromise patient and/or research staff confidentiality; these issues have now been resolved as described later in this paper.

## Data description

Women included in this study were at least 18 years old, over 20 weeks' gestation, with a live fetus and were scheduled to have an induced labour because of hypertensive disorders of pregnancy. Women unable to give informed consent, those with a previous caesarean section, multiple pregnancy, ruptured membranes, clinically diagnosed chorioamnionitis or a history of allergy to misoprostol were not recruited [4].

After informed consent, 602 women were randomly assigned to labour induction with oral misoprostol (25 µg every 2 h for a maximum of 12 doses) or a transcervical Foley catheter (size 18 F with 30 ml balloon). Randomisation schedules were computer-generated and administered using opaque sealed envelopes. Induction continued with artificial membrane rupture and oxytocin, administered through a micro-drip gravity infusion set [4].

Detailed information was collected on paper forms by research assistants at defined time points; immediately prior to IOL, at randomisation, every 2 h during the IOL process, at 24 h post-delivery and at discharge. Data were later double-entered into SPSS (IBM, Portsmouth, UK) by research staff in India and the US. The case report forms have been made publicly available along with a data dictionary and detailed description of methods (see Table 1).

**Table 1:** Overview of data files/data sets

Label	Name of data file/data set	File types (file extension)	Data repository and identifier (DOI or accession number)
Data file 1	INFORM database_CSV_160121	CSV data file (.csv)	UK Data Service ReShare http://doi.org/10.5255/UKDA-SN-854663 [7]
Data file 2	INFORM_database_Excel_060121	MS Excel file (.xlsx)	UK Data Service ReShare http://doi.org/10.5255/UKDA-SN-854663 [7]
Data file 3	INFORM_Data Dictionary_160121_sharing version	MS Excel file (.xlsx)	UK Data Service ReShare http://doi.org/10.5255/UKDA-SN-854663 [7]
Data file 4	Detailed_methods_INFORM_16NOV20.docx	MS Word file (.docx)	UK Data Service ReShare http://doi.org/10.5255/UKDA-SN-854663 [7]

Data on admission, induction and delivery includes basic demographic information, medical and obstetric history (current and previous), pre-eclampsia symptoms (severe nausea and vomiting, epigastric pain, headache, visual disturbance, chest pain or dyspnea), maternal and fetal observations and examination findings, detailed information on all medication administered, women’s expectations of pain and anxiety pre-IOL, side effects and complications of induction, mode of delivery and indication for delivery where relevant, complications and operative interventions. These data were used to calculate the number of vaginal births within specific timeframes (12, 24 h) and induction to birth intervals.

Postnatal data includes information on morbidity, complications, mortality, women’s rating of acceptability (regarding the amount of time taken and anxiety), whether women would recommend this method for future inductions, discharge dates and discharge medications.

Neonatal data include basic information such as birth outcome, birth weight, APGARs at 1, 5 and 10 min, age of first feed, gasp and heart rate over 100. Neonatal morbidity including diagnosis, SCBU admission and length of stay, oxygen administration, ventilation, seizures and age at first seizure, discharge/death date and discharge medication. Upon discharge, babies admitted to the special care unit were assessed for encephalopathy and the components of the original Sarnat score recorded [4].

Full details of the outcomes collected are included in the downloadable data dictionary and case report forms (see Table 1).

Due to concerns that including explicit dates (particularly date of delivery) could compromise anonymity, all dates have been replaced by number of days from a seed date prior to the start of this study; the identity of the seed date can be obtained on request, where appropriate.

## Limitations

This was a pragmatic study, based in busy Government Hospitals, where clinical decisions are typically made quickly, often without the full diagnostic work-ups and investigations required to differentiate between subgroups of hypertension in pregnancy (i.e. separation into proteinuric or non-proteinuric hypertension, or HELLP syndrome). Urinalysis was often not available, therefore making these results more generalizable to the whole population, but less focused on the treatment of hypertensive disorders specifically. Further investigations such as blood tests were not recorded.

This was an un-blinded study, due to ethical and practical concerns associated with undertaking unnecessary or sham Foley catheterisation. Whilst this risks bias, neither treatment was commonly used in the recruiting hospitals prior to this study and therefore clinician’s pre-existing views about treatment efficacy were unlikely to affect clinical decisions on these induction methods.

## Data Availability

The data described in this data note can be accessed on UK Data Service’s ReShare data repository under http://doi.org/10.5255/UKDA-SN-854663. Please see Table 1 and reference list [7] for details and links to the data.

## Abbreviations

INFORM: The Induction with Foley OR Misoprostol study
IOL: Induction of labour
HELLP syndrome: Haemolysis, elevated liver enzymes and low platelet count
